# Utility and safety of a new uneven double-lumen sphincterotome in cases of difficult biliary cannulation

**DOI:** 10.1186/s12876-021-01689-6

**Published:** 2021-03-04

**Authors:** Shuhei Shintani, Osamu Inatomi, Yoshiya Takeda, Hiroshi Matsumoto, Takehide Fujimoto, Yoshihisa Tsuji, Hiromu Kutsumi, Akira Andoh

**Affiliations:** 1 Department of Medicine, Shiga University of Medical Science, Seta Tsukinowa, Otsu, 520-2192 Japan; 2 Department of General Medicine, Sapporo Medical University, Sapporo, Japan; 3 Center for Clinical Research and Advanced Medicine, Shiga University of Medical Science, Otsu, Japan

**Keywords:** Sphincterotome, ERCP, Pancreatic guidewire method

## Abstract

**Background:**

We investigated the utility and safety of a new uneven double-lumen sphincterotome in biliary cannulation in comparison with the conventional pancreatic guidewire (PGW) method.

**Methods:**

We retrospectively evaluated 119 patients who required PGW placement because of difficult biliary cannulation. Endoscopic retrograde cholangiopancreatography (ERCP) was performed using a conventional ERCP catheter or a new uneven double-lumen sphincterotome. The success rate of bile duct cannulation, the operation time of bile duct cannulation, and the incidence of post-ERCP pancreatitis (PEP) were evaluated.

**Results:**

Forty-four patients were treated with a new double-lumen sphincterotome (the new sphincterotome group) and 75 patients underwent conventional PGW placement (the conventional group). The success rate of bile duct cannulation was 39/44 (88.6%) in the new sphincterotome group and 63/75 (84.0%) in the conventional group (not significant). The total biliary cannulation time (from the reach to the papilla to the finish of biliary cannulation) was 16.0 (6.5–78) min in the new sphincterotome group and 26.0 (5–80) min in the conventional group (*P* < 0.01). The time from PGW placement to bile duct cannulation was 3.5 (0.3–57) min in the magictome group and 12.0 (1–65) min in the conventional group (*P* < 0.01). Hyperamylasemia was observed in 13/44 (29.5%) and 17/75 (22.7%), respectively (not significant). Five of 44 (11.3%) of the new sphincterotome group and 14/75 (18.7%) of the conventional group were diagnosed with PEP (not significant).

**Conclusion:**

A new double-lumen sphincterotome allows selective bile duct cannulation to be performed in a shorter time than the conventional PGW method.

## Background

Endoscopic retrograde cholangiopancreatography (ERCP) is a standard technique for diagnosis and treatment of biliary disorders [1]. Various devices and techniques have been developed to improve the success rate of cannulation [1–3], but in some cases selective bile duct cannulation remains difficult due to papillary spasm and/or anatomical problem. Difficulty or failure of selective bile duct cannulation leads to a lengthening of procedure time and sometimes causes post-ERCP pancreatitis (PEP) that is occasionally fatal [4–6].

Pancreatic guidewire (PGW) placement has been reported to facilitate selective bile duct cannulation [1, 7]. PGW placement stabilizes the mobility of the papilla and linearize the distal part of the bile duct [3], leading to an increased success rate for selective bile duct cannulation. On the other hand, PGW placement has been reported to be associated with a risk of PEP due to contrast media injection and/or mechanical injury of the pancreatic duct [8]. PGW placement includes several steps: cannulation into the pancreatic duct to insert a guidewire, pull off the pancreatic cannula while retaining the guidewire, and cannulation or guidewire insertion into the bile duct [9]. These processes are somewhat complicated and time consuming. Moreover, placed PGW sometimes disturbs next approach to the common bile duct.

**Fig. 1:**
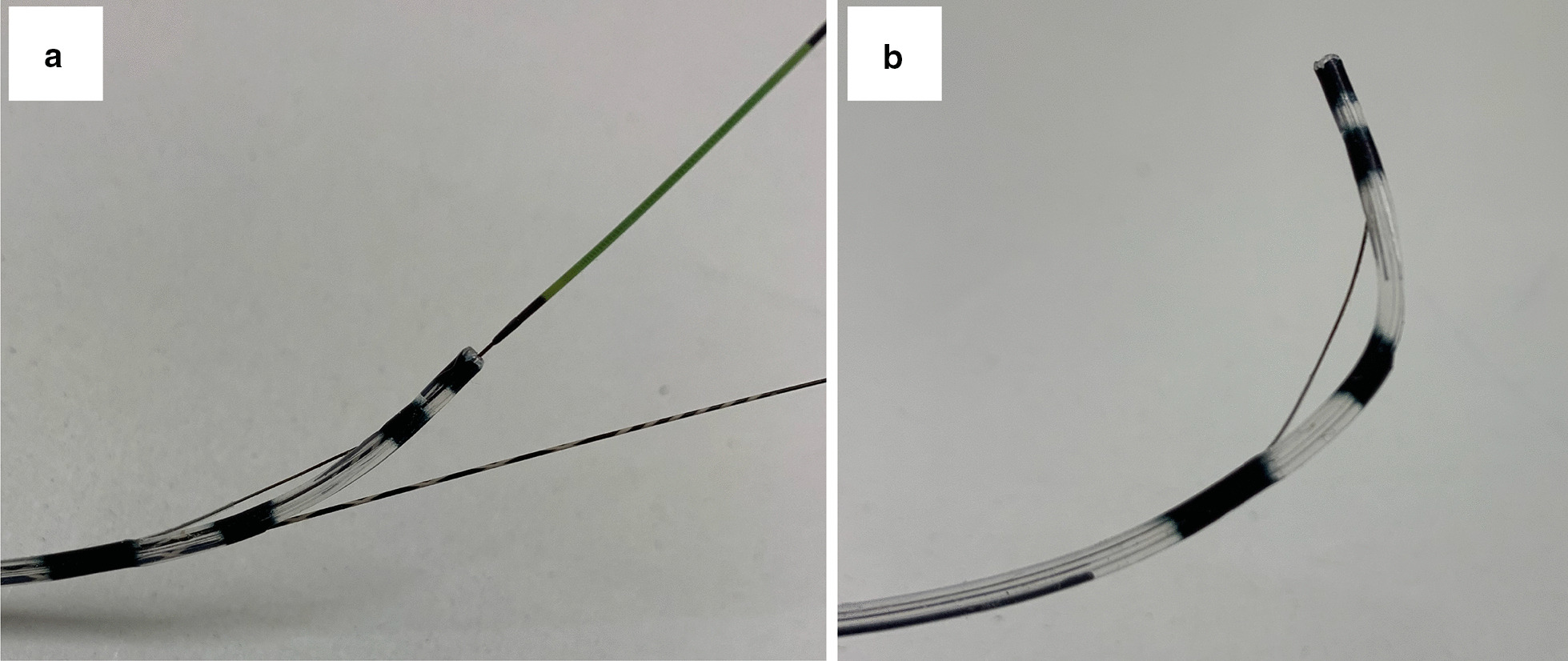
**a** MagicTome® (Piolax Inc., Yokohama, Japan) is a new double-lumen sphincterotome with distal (tip) and proximal (side) holes. **b** The angle of the device tip can be adjusted by raising the head

MagicTome® (Piolax Inc., Yokohama, Japan) is a new double-lumen sphincterotome with distal (tip) and proximal (side) holes (Fig. 1). Each lumen is 0.025 inch in diameter. The distance between the distal and proximal holes is 17 mm. This device allows PGW placement and bile duct cannulation without the need to change any devices. This may shorten total procedure time and improve the success rate of selective biliary cannulation. In 2018, we changed the cannulation method from conventional PGW placement using a single-lumen catheter to MagicTome®. In this study, we investigated whether MagicTome® reduces the operation time and alters the incidence of PEP after PGW placement.

## Methods

### Patients

From January 2013 to February 2020, 1,311 patients underwent ERCP at the Shiga University of Medical Science Hospital. Of these, we retrospectively evaluated 119 patients who required PGW placement because of difficult bile duct cannulation (a conventional cannulation into the bile duct needing more than 10 min or 10 unsuccessful attempts). ERCP was performed using a conventional ERCP catheter (MTW ERCP catheter: ABIS, Tokyo, Japan) from January 2013 to May 2018 (the conventional group) and the MagicTome® sphincterotome (Piolax) from June 2018 to February 2020 (the magictome group). Patients with a history of endoscopic sphincterotomy (EST), Billroth II surgery or pancreatic disease were excluded. All data related to ERCP was stored as a video and electronic database. All patients provided a written informed consent prior to undergoing ERCP. This study was approved by the ethics committee of the Shiga University of Medical Science (No. R2019-115). A trainee was defined as an operator who had performed fewer than 100 ERCP procedures within 5 years.

### Endoscopic procedure

A combination of midazolam and pentazocine were used for conscious sedation in all patients until 2014, and dexmethomidine was added from 2015. ERCP was conducted with a side-viewing duodenoscope JF260V (Olympus Optical Co., Tokyo, Japan) and an ERCP guidewire Visiglide2® (Olympus). A pancreatic duct stent (Geenen® 5Fr, Cook Medical, Tokyo, Japan) was applied if the operator required it in cases where EST was not performed. When performing the PGW placement, pancreatography was performed using a minimal amount of contrast injection. After confirming the clearance of contrast media from the pancreatic duct, a guidewire was inserted into the deep pancreatic duct. When PGW placement did not successfully achieve biliary cannulation, a precut sphincterotomy or another maneuver was performed.

### Selective bile duct cannulation using a double-lumen sphincterotome

**Fig. 2:**
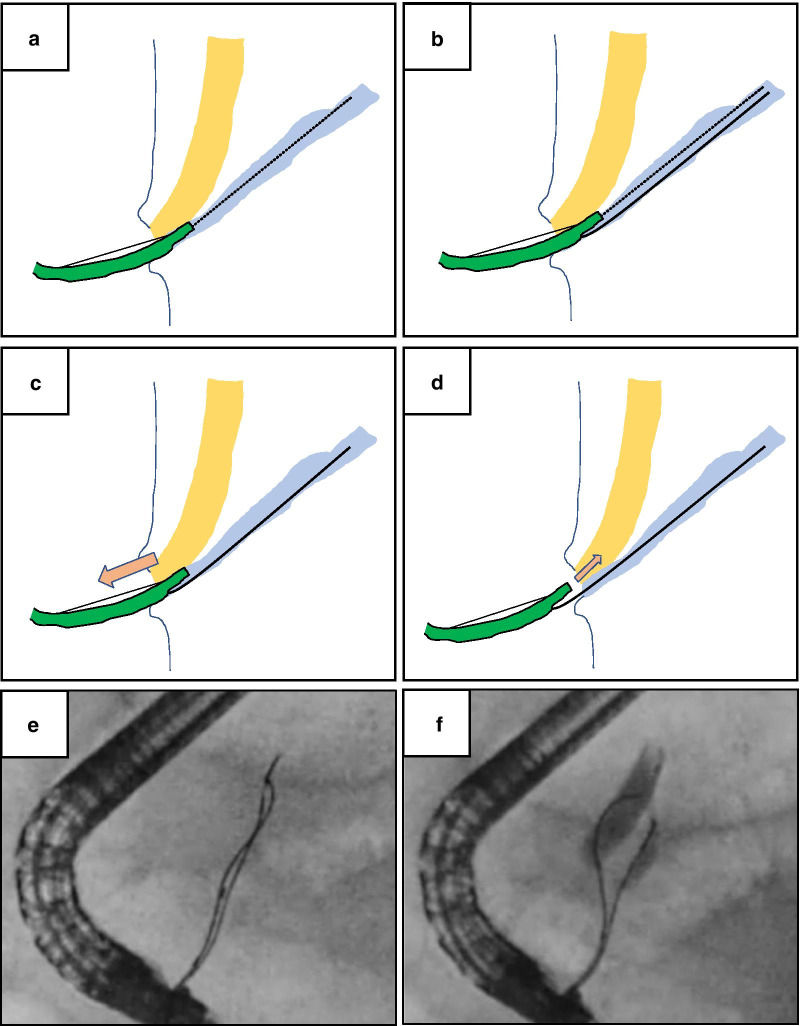
Process of selective bile duct cannulation using MagicTome®. **a** The guidewire is initially inserted into the pancreatic duct through the distal (tip) hole. **b** The catheter should be slightly put into the pancreatic duct, and another guidewire inserted into the pancreatic duct from the proximal (side) hole. **c** The guidewire inserted from the distal (tip) hole should be pulled into the catheter, and the catheter itself pulled back to the duodenum. **d** The approach to the papilla and bile duct can be performed from the duodenum. This method is characterized by not requiring the exchange or removal of any devices during the procedure. **e** Fluoroscopic image showing two guidewires in the pancreatic duct. **f** Fluoroscopic image showing successful bile duct cannulation

MagicTome® (Piolax) is a sphincterotomy knife with a double lumen at the tip and side holes. The pancreatic duct is fixed by the guidewire from the proximal (side) hole, and the bile duct and pancreatic duct are separated by raising the catheter tip. This enables subsequent bile duct cannulation from the distal (tip) hole. A schema of the typical process is presented in Fig. 2. (a) The operator inserts the guidewire into the pancreatic duct from the distal (tip) hole. (b) Then, the operator should slightly place the catheter into the pancreatic duct and insert another guidewire in the pancreatic duct from the proximal (side) hole. (c) The operator pulls the tip of the guidewire into the catheter, and then pulls the catheter back to the duodenum while retaining the pancreas guidewire. (d) The approach to the papilla and bile duct can be performed from the duodenum. This method does not require the exchange or removal of any devices during the procedure.

### Outcome

We investigated the success rate of bile duct cannulation, the operation time and the incidence of PEP of the biliary cannulation using MagicTome® in comparison with the conventional method using a single-lumen catheter.

### Safety evaluation

Hyperamylasemia, PEP, bleeding, perforation, and cholangitis were evaluated as complications. Diagnosis and disease severity of PEP were based on the criteria of Cotton et al. [4]. These include newly developed abdominal pain and elevation of serum amylase levels 3 times over the upper limit of normal level within 24 h [4].

### Statistical analysis

The continuous variables pertaining to the baseline characteristics of the two groups were compared by using the Student’s t-test or the Wilcoxon rank-sum test, as appropriate. Categorical variables were compared using the chi-square or Fisher’s exact test. Values of *p* < 0.05 were considered to be significantly different. Multivariate analysis was performed on factors that had *p* < 0.10 in the univariate analysis. All statistical analyses were performed using EZR version 1.40 (Jichi Medical University, Shimotsuke, Japan).

## Results

There were no significant differences in patients’ characteristics between the magictome group and the conventional group (Table 1).

**Table1:** Patients characteristics

	The magictome group	The conventional group	*P* value
	n = 44	n = 75	
Age, median (range), year	72.0 (42–89)	74.0 (36–93)	0.36
Sex (male/female), n	25 /19	48/27	0.56
Indication of ERCP, n (%)			
CBD stone	24 (54.5)	38 (50.7)	0.83
Neoplasm	16 (36.4)	22 (29.3)	0.56
Others	4 (9.1)	15 (20.0)	0.13
ASA ≥ 3, n (%)	4 (9.1)	9 (12.0)	0.77
Anticoagulant use, n (%)	7 (15.9)	17 (22.7)	0.52
Duodenum diverticulum, n (%)	3 (6.8)	5 (6.7)	1.0
Oral protrusion of the papilla, mm (mean ± SD)	10.4 ± 4.9	9.7 ± 2.9	0.33
Purpose of ERCP, n (%)			
EST	27 (61.4)	39 (52.0)	0.42
EPBD	1 (2.3)	6 (8.0)	0.26
IDUS	15 (34.1)	16 (21.3)	0.19
EBD	20 (45.5)	31 (41.3)	0.81
Biopsy	2 (4.5)	3 (4.0)	1.0
Cases performed by trainee, n (%)	22 (50.0)	32 (42.7)	0.56

CBD, common bile duct; ASA, American Society of Anesthesia Classification (Owens WD, et al. Anesthesiology 1978, 49:239–243.); EST, endoscopic sphincterotomy; EPBD, endoscopic papillary balloon dilation; IDUS, intraductal ultrasound sonography; EBD, endoscopic biliary drainage

**Fig. 3:**
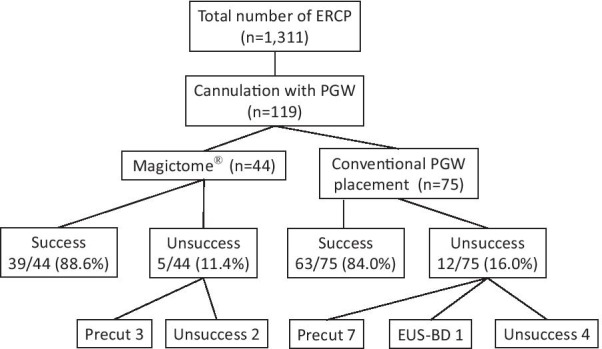
Flowchart showing the treatment course of the patients enrolled in this study. ERCP: endoscopic retrograde cholangiopancreatography, PGW: pancreatic duct guidewire, precut: precut sphincterotomy, EUS-BD: endoscopic ultrasound biliary drainage

Of 119 patients who underwent PGW placement, 44 patients were treated with MagicTome® (the magictome group) and 75 patients underwent conventional PGW placement (the conventional group) (Fig. 3). The success rate of bile duct cannulation was 39/44 (88.6%) in the magictome group and 63/75 (84.0%) in the conventional group (no significant difference) (Table 2). Of 5 patients with bile duct cannulation failure in the magictome group, 3 patients subsequently received precut sphincterotomy, but bile duct cannulation was a failure in 2 patients. In the conventional group, bile duct cannulation was unsuccessful in 12 patients, and 7 patients were successfully cannulated after precut sphincterotomy (Fig. 3).

**Table2:** Outcome and complications

	The magictome group	The conventional group	*P* value
	n = 44	n = 75	
Successful biliary cannulation, n (%)	39 (88.6)	63 (84.0)	0.67
Procedure time, min, median (range)			
Total biliary cannulation time^a^	16.0 (6.5–78)	26.0 (5–80)	< 0.01
Time from PGW placement to biliary cannulation	3.5 (0.3–57)	12.0 (1–65)	< 0.01
Complications, n (%)			
Hyperamylasemia	13 (29.5)	17 (22.7)	0.54
PEP	5 (11.4)	14 (18.7)	0.44
Cholangitis	3 (6.8)	2 (2.7)	0.36
Bleeding	1 (2.3)	4 (5.3)	0.65
Perforation	0	1 (1.3)	1.0
Placement of pancreatic duct stent, n (%)	10 (22.7)	18 (24.0)	1.0

PEP, post-ERCP pancreatitis

^a^From the reach to the papilla to the finish of biliary cannulation

The total biliary cannulation time (from the reach to the papilla to the finish of bile duct cannulation) was 16.0 (6.5–78) min in the magictome group and 26.0 (5–80) min in the conventional group (*P* < 0.01) (Table 2). The time from PGW placement to biliary cannulation was 3.5 (0.3–57) min in the magictome group and 12.0 (1–65) min in the conventional group (*P* < 0.01).

Placement of a pancreatic duct stent was performed in 10/44 (22.7%) in the magictome group and 18/75 (24.0%) in the conventional group (no significant difference). Hyperamylasemia was observed in 13/44 (29.5%) of the magictome group and 17/75 (22.7%) in the conventional group (no significant difference) (Table 2). Five of 44 (11.3%) of the magictome group and 14/75 (18.7%) of the conventional group were diagnosed with PEP (no significant difference) (Table 2).

## Discussion

This study revealed a number of important points regarding the use of MagicTome® in selective bile duct cannulation. While MagicTome® significantly shortened the bile duct cannulation time, it did not alter the success rate of the procedure. Complication rates associated with MagicTome® use were comparable to those of the conventional PGW placement.

PGW placement, such as the contrast-medium method and the contrast-free guidewire cannulation method, has been reported to be useful for selective biliary cannulation in patients in whom biliary cannulation is difficult [10]. The PGW method was first reported in 1998 by Dumonceau et al. as useful for biliary cannulation in Billroth I gastrectomy cases [11]. Gotoh et al. reported the usefulness of the double-guidewire method to straighten a tortuous pancreatic duct [12]. Ito et al. reported that 82 out of 113 (72.6%) patients with difficulties in biliary cannulation were successfully cannulated by PGW placement [10]. However, PGW placement requires the removal of the pancreas catheter while retaining the guidewire left in the pancreatic duct. This step is inconvenient and time-consuming. The placed PGW occasionally disturbs next approach to the common bile duct. Moreover, it is sometimes difficult to insert the catheter or guidewire in the direction of the bile duct, which delays biliary cannulation.

In a novel approach, Takenaka et al. recently reported the usefulness of a newly-developed biliary cannulation method for difficult cannulation cases using a unique, uneven, double-lumen cannula (uneven method) [2, 13]. They reported that their cannula straightened the pancreatic duct and the common channel, thereby effectively stabilizing the papilla and enabling easy adjustment of the catheter axis to comply with the bile duct direction.

In this study, we reported a new bile duct cannulation method for difficult cannulation cases using a MagicTome® sphincterotome. Similar to the uneven cannula used by Takenaka et al., MagicTome® is a uneven double-lumen sphincterotome with distal and proximal holes. However, there are several differences in the method of bile duct cannulation between the uneven cannula and MagicTome®. PGW placement using the uneven cannula is initiated via the distal (tip) hole, whereas using the MagicTome® it is placed via the proximal (lateral) hole. The tip of the uneven cannula is inserted and fixed in the pancreatic duct, but MagicTome® can move freely and allows free approach to the papilla or common bile duct. When needed, precut sphincterotomy is easily performed with MagicTome® without any device exchange. Thus, there are a number of differences in bile duct cannulation approach between the uneven cannula and MagicTome®.

The success rate of bile duct cannulation tended to be higher in the magictome group (88.6%) than in the conventional group (84%), but there was no significant difference. The current study was performed in cases of difficult biliary cannulation, and the successful cannulation rate in this study is acceptable when comparing with previous reports [9, 14]. This issue should be prospectively reconfirmed in a multicenter study with much greater number of patients. Both total biliary cannulation time and the time from PGW placement to bile duct cannulation were significantly reduced in the magictome group compared to the conventional group. These findings indicate that MagicTome® use is effective in shortening the operation time for selective bile duct cannulation in patients where biliary cannulation is difficult. This may be associated with the fact that MagicTome® needs no device exchange. Previously reported effects of PGW placement such as opening a stenotic papillary orifice, stabilizing the papilla and straightening the pancreatic duct and the common channel might be also involved [14]. In addition, its ability to easily adjust its axis to comply with the bile duct direction might contribute to the shortening of operation time. Shortening the operation time may benefit not only the risk of PEP but also complication associated with sedation and radiation exposure of the operators.

Precut sphincterotomy seemed to be ineffective in the magictome group compared to the conventional group. Although the number of patients with unsuccessful biliary cannulation in the magictome group was small (n = 3), such patients might have anatomical constraints that make cannulation particularly difficult.

Many studies reported prophylactic effect of pancreatic duct stent placement on PEP [15, 16]. However, the benefit remains still controversial in terms of the costs and risks associated with approaching the pancreatic duct [17]. Therefore, in the present study, placement of pancreatic duct stent was performed in a limited number of patients. Information bias was minimal as the frequency was comparable between two groups.

The safety of MagicTome® use was confirmed to some extent, since there were no significant differences in the incidence of hyperamylasemia and PEP between the magictome group and the conventional group. Pancreatitis remains the most common complication of ERCP, occurring in 4% to 7% of unselected patients to 30% in high-risk patients [8, 18, 19]. The edema of the papilla caused by repeated cannulation and/or the increased pressure from contrast injection are risk factors for PEP [19]. The influence of PGW placement on PEP remains controversial. PGW placement has been reported to improve the success rate of bile duct cannulation, reduce papillary trauma, and avoid inadvertent contrast injection, thereby reducing the risk of PEP [19]. On the other hand, mechanical injury of the pancreatic duct by PGW has been reported to be a potential risk factor [19], suggesting that the shortening of retaining time of PGW using MagicTome® may reduce the incidence of PEP.

## Conclusions

The MagicTome® technique is a feasible and safe method in difficult bile duct cannulation avoiding the complicated processes that occur in the conventional PGW method. This was a retrospective, single-center study comparing MagicTome® use with the conventional technique. As such, the usefulness and safety of MagicTome® including the issues other than those assessed in this study should be investigated in a prospective randomized study in the future.

## Data Availability

The datasets used during the current study available from the corresponding author on reasonable request.

## Abbreviations

ERCP: Endoscopic retrograde cholangiopancreatography
PGW: Pancreatic guidewire
PEP: Post-ERCP pancreatitis
precut: Precut sphincterotomy
EUS-BD: Endoscopic ultrasound biliary drainage
CBD: Common bile duct
ASA: American Society of Anesthesia Classification
EST: Endoscopic sphincterotomy
EPBD: Endoscopic papillary balloon dilation
IDUS: Intraductal ultrasound sonography
EBD: Endoscopic biliary drainage
